# RETRACTION: Pathogenesis of Psoriasis via MiR‐149‐5p/AKT1 Axis by Long Noncoding RNA BLACAT1

**DOI:** 10.1111/srt.70219

**Published:** 2025-08-25

Hua X, Li JZ, Shang MW, et al. Pathogenesis of psoriasis via miR‐149‐5p/AKT1axis by long noncoding RNA BLACAT1. *Skin Res Technol*. 2023;29(5):e13339. doi:10.1111/srt.13339

The above article, published online on 10 May 2023 in Wiley Online Library (wileyonlinelibrary.com), has been retracted by agreement between *Skin Research and Technology*; and John Wiley & Sons Ltd. The article has been accepted for publication following an inadequate peer review process. Following publication, it has come to the attention of the journal that the ethical approval granted for the study does not explicitly authorize the collection of normal skin tissues from healthy volunteers. Furthermore, flaws and inconsistencies between methodology described and results presented were identified. The authors did not sufficiently address the identified concerns. Accordingly, the article is retracted. The authors have been informed of the decision of retraction and disagree with it.

